# Childcare burden and changes in fertility desires of mothers during the COVID-19 pandemic

**DOI:** 10.3389/fpsyg.2023.1243907

**Published:** 2023-11-30

**Authors:** Kateryna Golovina, Ilona Nenko, Urszula Maria Marcinkowska

**Affiliations:** ^1^Helsinki Collegium for Advanced Studies, University of Helsinki, Helsinki, Finland; ^2^Faculty of Health Sciences, Jagiellonian University Medical College, Kraków, Poland

**Keywords:** childcare, equality, pandemic, reproduction, women

## Abstract

**Objectives:**

Previous studies have documented a decline in fertility desires and intentions following the COVID-19 outbreak, but the reasons for this decline are not well understood. This study examined whether childcare burden on mothers during the lockdown and quarantines, COVID-related stress, and COVID exposure were associated with a change in the desired number of children.

**Methods:**

The survey was conducted online, in Poland from April to July 2021 on a sample of 622 non-pregnant mothers without diagnosed infertility.

**Results:**

Associations were observed between childcare responsibilities during the quarantine and fertility desires: mothers who solely or mainly took care of their children during the quarantine(s) were more likely to decrease their desired number of children ([adjusted] aOR = 1.91, 95% CI = 1.16–3.15). Mothers with higher levels of COVID-related stress (aOR = 1.81, 95% CI = 1.48–2.22) and a greater COVID exposure index (aOR = 1.39, 95% CI = 1.12–1.72) were more likely to decrease their fertility desires.

**Conclusion:**

Higher childcare burden during quarantines was related to a lower desired number of children among mothers. Both greater COVID-related stress and COVID exposure were associated with fertility desires, regardless of childcare responsibilities during the pandemic.

## Introduction

Following the COVID-19 outbreak, numerous studies have shown a decline in birth rates in high-income countries (Aassve et al., 2021; Pomar et al., 2022; Wang et al., 2022), although some differences between countries and regions were observed (Cohen, 2021; Nitsche et al., 2022). Fertility preferences (i.e., an umbrella term for fertility desires, intentions, expectations, and plans) also changed because of the COVID-19 pandemic: most people delayed or abandoned their fertility plans during the pandemic, as shown by a recent review (Safdari Dehcheshmeh et al., 2023). Among the factors related to changes in fertility preferences during the COVID-19 pandemic, previous studies highlighted economic conditions (e.g., job insecurity, unemployment, decreased income, or fear of income decline) (Lindberg et al., 2020; Micelli et al., 2020; Arpino et al., 2021; Kahn et al., 2021; Lin et al., 2021; Malicka et al., 2021; Sienicka et al., 2021; Tan et al., 2021), access to healthcare services (Flynn et al., 2021; Sienicka et al., 2021), health concerns (e.g., worries about the effect of the virus on pregnant women and unborn children) (Micelli et al., 2020; Flynn et al., 2021), and increased psychological distress, anxiety, and depressive symptoms. (Kahn et al., 2021; Malicka et al., 2021; Marteleto and Dondero, 2021; Naya et al., 2021; Tan et al., 2021). Increased childcare burden on parents during lockdowns was also suggested to result in lower fertility desires (Aassve et al., 2020), but research on this is scarce. By examining factors that contribute to the decline in fertility desires during crises, such as the COVID-19 pandemic, and especially focusing on mothers, we improve our understanding of the decision to have another child during times of uncertainty. This information may be especially relevant for policymakers to help mitigate the effects of crises on people’s fertility plans. Therefore, our study examined the role of childcare burden, socio-demographic characteristics, as well as COVID-19 exposure and COVID-related stress in the fertility desires of mothers.

### Previous research on fertility desires during the COVID-19 pandemic

Previous research indicated a change in fertility preferences during the COVID-19 pandemic, with people tending to postpone or abandon their fertility plans (Safdari Dehcheshmeh et al., 2023). Although there were some differences between countries due to their economic and social conditions (Luppi et al., 2020), the overall tendency to delay childbearing was evident in all countries under study. Most of the previous studies examined changes in fertility intentions during the COVID-19 pandemic (Luppi et al., 2020; Zhu et al., 2020; Arpino et al., 2021; Flynn et al., 2021; Kahn et al., 2021; Lin et al., 2021; Malicka et al., 2021; Marteleto and Dondero, 2021; Sienicka et al., 2021; Tan et al., 2021; Buber-Ennser et al., 2023), and only a few studies investigated changes in fertility desires (Lindberg et al., 2021; Lazzari et al., 2023). Fertility desires represent individuals’ explicit wishes regarding childbearing (i.e., the preferred number of children and timing for childbearing), whereas fertility intentions are defined as more concrete plans for childbearing considering current circumstances and obstacles (Miller, 2011; Philipov and Bernardi, 2012). Changes in short-term fertility intentions were especially pronounced at the beginning of the COVID-19 pandemic (Micelli et al., 2020; Flynn et al., 2021; Kahn et al., 2021; Naya et al., 2021), whereas changes in fertility desires or long-term fertility intentions were moderate (Malicka et al., 2021; Lazzari et al., 2023) or even minimal (Emery and Koops, 2022; Buber-Ennser et al., 2023). Finally, several studies also showed that there was a small group of people reporting increased fertility preferences (Micelli et al., 2020; Lindberg et al., 2021; Tan et al., 2021; Lazzari et al., 2023) or a desire to accelerate their childbearing (Flynn et al., 2021; Lindberg et al., 2021; Naya et al., 2021) because of the pandemic.

At the beginning of the COVID-19 pandemic, changes in fertility intentions among women who were trying to conceive were very pronounced, with up to 50% of them delaying or abandoning their plans because of it (Micelli et al., 2020; Flynn et al., 2021; Kahn et al., 2021; Naya et al., 2021). However, the impacts of the pandemic on fertility plans reported in the summer of 2021 were smaller, with about 20% of women reporting changes in their fertility plans (Lindberg et al., 2021). Among couples who intended to have children before the COVID-19 pandemic, about one-third canceled their fertility plans because of it (Micelli et al., 2020; Zhu et al., 2020). Also, among infertile couples seeking assisted reproductive therapy, there was a noticeable increase in psychological distress and negative emotions due to the COVID-19 pandemic (Irani et al., 2022). Most of the previous studies included only women (Flynn et al., 2021; Lin et al., 2021; Marteleto and Dondero, 2021; Naya et al., 2021; Afshari et al., 2022) or both men and women (Luppi et al., 2020; Arpino et al., 2021; Malicka et al., 2021; Sienicka et al., 2021; Emery and Koops, 2022), and did not focus specifically on parents [with several exceptions (Kahn et al., 2021; Malicka et al., 2021; Lazzari et al., 2023)]. However, there is evidence that parents with one or two children were more likely to revise their fertility desires and intentions than childless individuals (Malicka et al., 2021; Lazzari et al., 2023). This might be related to increased childcare burden on parents during lockdowns and quarantines or elevated parental stress, although this was not directly examined in previous studies.

### Factors related to changes in fertility desires among parents during the COVID-19 pandemic

Aassve et al. (2020) suggested that an increased childcare burden on parents due to closed childcare centers and schools during lockdowns might result in lower fertility desires and postponement of childbearing in the short run. Additionally, the division of childcare and housework responsibilities during lockdowns might affect fertility desires and subsequent behavior (Aassve et al., 2020). According to the gender equity framework, men’s involvement in domestic work is related to a reduced burden on women for childcare and housework, resulting in more positive fertility preferences (Goldscheider et al., 2015). Although some have argued that the rise of telecommuting would promote higher equality in domestic childcare and housework (Mas and Pallais, 2020), recent studies from the US and Italy found little evidence for a reduced gender gap in domestic work during the pandemic (Del Boca et al., 2020; Dunatchik et al., 2021).

Furthermore, increased anxiety and stress related to the COVID-19 pandemic might also be related to the decline in fertility desires, as well as amplify the relationship between childcare burden and fertility desires. Given that fertility usually declines during times of crisis (Aassve et al., 2020), the high prevalence of anxiety symptoms and fear about the consequences of the pandemic might result in the decision to postpone childbearing, especially among women (Alsharawy et al., 2021; Pashazadeh Kan et al., 2021). It is also possible that concerns about the potential effects of severe acute respiratory syndrome coronavirus-2 (SARS-CoV-2) infection on the fetus during pregnancy might additionally be related to the decision to postpone childbearing (Micelli et al., 2020; Zhu et al., 2020; Flynn et al., 2021). Moreover, it is known that parental stress is associated with fertility decline (Li et al., 2020), so it is likely that even despite a low childcare burden or more or less equal share of responsibilities between parents, higher levels of stress might be related to decreased fertility desires.

The majority of previous studies also highlighted the role of socio-economic conditions in the decline of fertility preferences (Lindberg et al., 2020; Micelli et al., 2020; Arpino et al., 2021; Kahn et al., 2021; Lin et al., 2021; Malicka et al., 2021; Sienicka et al., 2021; Tan et al., 2021). For example, low-income women or those whose income has changed because of the pandemic were more likely to reduce their fertility plans (Lindberg et al., 2020; Kahn et al., 2021; Lin et al., 2021; Tan et al., 2021). Also, those individuals who expected an insecure future income due to the pandemic were more likely to modify their fertility plans (Luppi et al., 2020; Arpino et al., 2021; Sienicka et al., 2021). Lastly, non-employed women had more negative attitudes toward fertility during the pandemic (Arpino et al., 2021; Afshari et al., 2022).

### Present study

This study contributes to the previous literature by examining factors related to the decline in fertility desires during the COVID-19 pandemic, focusing specifically on mothers using survey data from Poland. Given that women faced more difficulties when combining work and childcare responsibilities (Del Boca et al., 2020), we studied women who already had children when the pandemic began. Our first aim was to examine the association between childcare burden of mothers during the lockdown and quarantines during the COVID-19 pandemic with fertility desires. Based on the gender equity framework (Goldscheider et al., 2015; Aassve et al., 2020) suggestion about the increased childcare burden on parents during the COVID-19 pandemic, we hypothesized that greater childcare burden on mothers during the pandemic would be related to a decline in fertility desires. Our second aim was to investigate whether exposure to the SARS-CoV-2 infection and COVID-related stress were related to changes in fertility desires of mothers. Our third aim was to investigate whether COVID-related stress moderated the relationship between childcare burden and fertility desires. Based on previous studies (Li et al., 2020; Zhu et al., 2020; Afshari et al., 2022), we hypothesized that COVID-19 exposure and higher COVID-related stress will be associated with decreased fertility desires. Finally, we also studied whether mothers whose fertility desires changed because of the pandemic differed in socio-demographic characteristics compared to mothers whose fertility desires did not change. In line with previous studies (Lindberg et al., 2020; Luppi et al., 2020; Kahn et al., 2021), we expect that less financially secure mothers will be more likely to decrease their fertility desires.

## Methods

### Sample

The sample was recruited via an online survey posted on the Qualtrics Platform and promoted in online groups and social media between April and July 2021 (pandemic-related restrictions were introduced in Poland in March 2020). The total sample comprised 2,639 men and women aged 18 to 65. For our study purposes, we included all women aged 18–45 who had at least one child and no diagnosed infertility (*n* = 801). Women who were pregnant at the time of data collection (*n* = 102) were excluded from the sample. We also further excluded women who identified themselves as exclusively or predominantly homosexual, or asexual (*n* = 7) because minority sexual orientation is associated with lower fertility desires and intentions (Baiocco and Laghi, 2013; Vseviov et al., 2023). Finally, those mothers who did not report their fertility desires (*n* = 29), COVID-19 exposure and stress (*n* = 14), and childcare burden (*n* = 27) were excluded, therefore the final analytical sample resulted in 622 women.

Ethics approval of the study protocol was granted by the Bioethics Committee at Jagiellonian University (opinion number 1072.6120325.2020, obtained on 25.11.2020) and consent was obtained from all participants. The study was conducted according to the highest ethical standards as described in the Declaration of Helsinki and the Oviedo Bioethics Convention.

### Measures

*Fertility desires*: All women were asked how many children they wanted to have before the pandemic and then during the time of the interview in April – July 2021. The change score in fertility desires was computed by subtracting the desired number of children before the pandemic from the desired number of children during the time of the interview. We further categorized the change score into a score characterizing a decline in a desired number of children: 0 = *remaining the same or increasing* [because few mothers reported an increase in the desired number of children (*n* = 31, 5.0%), 1 = *declining*].

*Childcare burden* was measured during the lockdown as well as during the quarantine with children. First, for the overall childcare burden in lockdown, all mothers were asked who took care of the children during the day in lockdown. The variable was coded in the following way: 1 = *only/mainly me*, 2 = *half-time me and my partner*, 3 = *only/mostly my partner/someone else.* Second, the mothers were asked whether they were in quarantine with their children, and those who responded positively were further asked who took care of the children during the day in quarantine (0 = *not in quarantine with children*, 1 = *only/mainly me*, 2 = *half-time me and my partner*, 3 = *only/mostly my partner/someone else*).

COVID *exposure index* was measured with four questions: (1) whether the participants ever had a positive SARS-CoV-2 test result, (2) whether they had contact with a person infected with the SARS-CoV-2 virus, (3) whether any of the participant’s family members had COVID-19, and (4) whether any of the participant’s friends or acquaintances had COVID-19. All responses were coded as 0 = *no* and 1 = *yes* and summed to form a COVID exposure index (0 = *no exposure*, 4 = *exposure to all four factors*). Given that very few participants were exposed to all four items, the COVID exposure index was coded in the following way: 0 = *no exposure* to 3 = *exposure to 3 or 4 factors*.

*COVID-related stress* was measured with a scale comprising six statements about COVID-related experiences (Shevlin et al., 2020) (e.g., “I worry a lot since the COVID-19 pandemic started”; see Appendix Table 1 for a full overview of the scale). Participants were asked to indicate how much they agreed with the statements using a 5-point Likert scale (1 = *not at all*, 5 = *almost all the time*). The participants were included in the analyses if they had no more than three missing items on the COVID-related stress scale. The mean score of these items was computed and used in the analyses. The scale was further divided into quartiles to test for a possible dose–response association with fertility desires. The internal consistency of the scale was good, with Cronbach’s α = 0.89. Based on the results of the confirmatory factor analysis, the construct validity was also good (see Appendix Table 2 for details).

*Socio-demographic characteristics* included age, the number of children, partnership status, education, employment situation, a self-reported judgment of own financial situation, place of residence, and change in income during the lockdown. Age was used as a continuous variable in the analysis, and the number of children was coded as 1, 2, or 3 or more children. The partnership status was coded in the following way: 1 = *single* (included participants who were single or not in stable relationships, as well as those who were divorced, separated, or widowed), 2 = *in a stable relationship*, and 3 = *married.* Education was coded as 1 = *secondary or lower* (comprised participants with basic, professional, or medium education), 2 = *lower tertiary* (bachelor’s degree), and 3 = *upper tertiary* (master’s degree or doctorate). Employment situation was coded as 1 = *not studying nor working*, 2 = *studying, or working and studying*, and 3 = *working*. The judgment of own financial situation was asked with the question “How would you describe your financial situation?” with 5 response options ranging from 1 = *very bad* to 5 = *very good*. Due to the small number of participants rating their financial situation as very bad or bad (*n* = 9, 1.4%), the variable was coded in the following way: 1 = *very bad/bad/average*, 2 = *good*, and 3 = *very good*. Change in income during the lockdown was asked with the question “Has the pandemic (ongoing lockdown) affected your income?” and coded as 0 = *no*, 1 = *yes, decreased*, and 2 = *yes, increased*. Finally, the place of residence was coded as a categorical variable in the following way: 1 = *village*, 2 = *<20,000–100,000 residents*, 3 = *100,000–500,000 residents*, and 4 = *>500,000 residents*.

### Statistical analysis

First, we compared the socio-demographic characteristics of mothers who decreased their desired number of children to those who did not change them using χ^2^ tests and independent sample two-tailed t-test. The socio-demographic characteristics included age, partnership status, the number of children, education, employment situation, judgment of own financial situation, place of residence, and change in income during the pandemic. We then examined the associations between these socio-demographic characteristics and a decline in the desired number of children using multivariable logistic regression models.

Second, to examine the associations between childcare burden and a decline in the desired number of children, we fitted simple logistic regression models to estimate crude associations and multivariable models to estimate adjusted associations. The adjusted models included all the above-mentioned socio-demographic characteristics. The models were further adjusted for COVID-related stress to examine whether the childcare burden was related to the decline in the desired number of children over and above COVID-related stress. We then examined the interactions between COVID-related stress and childcare burden on a decline in the desired number of children in the fully adjusted model. Age and COVID-related stress were mean-centered to facilitate the interpretation of the estimates. Finally, we also examined whether the COVID exposure index was associated with the decline in the desired number of children.

All analyses were conducted in Stata 17.0 (StataCorp LLC, 2021).

## Results

### Descriptive statistics

Table 1 shows the characteristics of the sample. The mean age of mothers was 34.5 years (SD = 4.49), 84.4% were married, and 50.6% had one child, followed by 36.7% having two children, and 12.7% having three or more children. Most of the women were highly educated (75.4% had a master’s degree or higher) and employed (74.0%). Half of the sample came from large cities with over 500,000 residents. About one-third of the mothers decreased their desired number of children due to the COVID-19 pandemic (28.9%), whereas 5.0% increased their desired number of children and 66.1% did not change their fertility desires. Overall, 31.7% of mothers reported being in quarantine with children, and 22.4% of them did not have any help with childcare.

**Table 1:** Descriptive statistics (*n* = 622).

Variables	*n* (%)
*Age, Mean (SD), ranged 19–45*	34.5 (4.49)
*Partnership status*	
Single	17 (2.7%)
In a stable relationship	80 (12.9%)
Married	525 (84.4%)
*Number of children*	
One	315 (50.6%)
Two	228 (36.7%)
Three or more	79 (12.7%)
*Education*	
Basic/professional/medium	68 (10.9%)
Bachelor’s degree	85 (13.7%)
Master’s/doctoral degree	469 (75.4%)
*Employment situation*	
Not studying or working	107 (17.2%)
Working and/or studying	55 (8.8%)
Working	460 (74.0%)
*Own financial situation*	
Bad/average	170 (27.3%)
Good	328 (52.7%)
Very good	124 (20.0%)
*Change in income during the pandemic*	
No	403 (64.8%)
Yes, decreased	183 (29.4%)
Yes, increased	36 (5.8%)
*Place of residence*	
Village	101 (16.2%)
<20,000–100,000 residents	114 (18.3%)
100,000–500,000 residents	90 (14.5%)
>500,000 residents	317 (51.0%)
*Change in a desired number of children because of the pandemic*	
Remained the same	411 (66.1%)
Decreased	180 (28.9%)
Increased	31 (5.0%)
*Taking care of children during the lockdown*	
Only/mainly me	346 (55.6%)
Half-time me and my partner	179 (28.8%)
Only/mostly my partner or someone else	97 (15.6%)
*Taking care of children during the quarantine*	
Not in quarantine with children	425 (68.3%)
Only/mainly me	95 (15.3%)
Half-time me and my partner	96 (15.4%)
Only/mostly my partner or someone else	6 (1.0%)
*COVID exposure index, Mean (SD), ranged 0–3 for a sum score*	2.08 (0.89)
0 (no exposure)	34 (5.5%)
1	122 (19.7%)
2	226 (36.4%)
3 (exposure to 3 or 4 items)	238 (38.4%)
*COVID-related stress, Mean (SD), ranged 1–5 for a mean score*	2.53 (0.95)
0 (lowest quartile)	146 (23.5%)
1	127 (20.4%)
2	181 (29.1%)
3 (highest quartile)	168 (27.0%)

Numbers (percentages) are reported unless otherwise indicated.

Mothers who decreased their fertility desires were more likely to have one child compared to those who did not change their fertility desires (59.4% vs. 47.1%, *p* = 0.014; Table 2). They were more likely to study or to combine work with studies (13.9% vs. 6.8%, *p* = 0.018), had a worse financial situation (34.4% vs. 24.4% for an average or lower financial situation *p* = 0.013), and their income declined during the pandemic (41.7% vs. 24.4%, *p* < 0.001) compared to mothers who did not change their fertility desires (Table 2). In line with the results of the *χ*^2^ tests, findings from the multivariable logistic regression analysis show that mothers with two or more children were less likely to decrease their fertility desires compared to mothers with one child after adjusting for the rest of the control variables (Appendix Table 3). Likewise, mothers who were studying or combining work with studies were more likely to decrease their fertility desires compared to mothers who were not studying nor working or who were only working. Additionally, mothers whose income declined during the pandemic were more likely to decrease their fertility desires compared to mothers whose income did not change during the pandemic (Appendix Table 3).

**Table 2:** The socio-demographic characteristics of mothers who decreased their fertility desires vs. those who did not change them.

	Desired number of children		
Socio-demographic characteristics	Not changed	Decreased	Value of *p*
*Age (Mean)*	34.6	34.2	0.371*
*Partnership status*			0.532
Single/not in a stable relationship	3.2%	1.7%	
In a stable relationship	12.4%	13.9%	
Married	84.4%	84.4%	
*Number of children*			**0.014**
One	47.1%	59.4%	
Two	38.7%	31.7%	
Three or more	14.3%	8.9%	
*Education*			0.184
Basic/professional/medium	9.5%	14.4%	
Bachelor’s degree	14.3%	12.2%	
Master’s / doctoral degree	76.2%	73.3%	
*Employment situation*			**0.018**
Not studying or working	17.9%	15.6%	
Working and/or studying	6.8%	13.9%	
Working	75.3%	70.6%	
*Own financial situation*			**0.013**
Bad/average	24.4%	34.4%	
Good	53.4%	51.1%	
Very good	22.2%	14.4%	
*Change in income during the pandemic*			**<0.001**
No	69.7%	52.8%	
Yes, decreased	24.4%	41.7%	
Yes, increased	5.9%	5.6%	
*Place of residence*			0.462
Village	16.5%	15.6%	
<20,000–100,000 residents	16.7%	22.2%	
100,000–500,00 residents	14.7%	13.9%	
>500,000 residents	52.0%	48.3%	

*p*-values for differences in categorical variables between the two groups were from the *χ*^2^ tests.

*p-value for differences in age between the two groups was derived from the independent sample two-tailed *t*-test.

### Associations between childcare burden and desired number of children

Table 3 shows the associations between the childcare burden during the COVID-19 pandemic and the decline in fertility desires among mothers. No associations were found between childcare responsibilities during the lockdown and the decline in fertility desires (both in crude and adjusted models). In contrast, associations were observed between childcare responsibilities during the quarantine and changes in fertility desires: mothers who solely or mainly took care of their children during the quarantine were more likely to decrease their desired number of children (adjusted OR = 1.91, 95% CI 1.16, 3.15; Model 1). When the model was additionally adjusted for COVID-related stress, this association decreased (adjusted OR = 1.78, 95% CI 1.06, 2.99; Model 2).

**Table 3:** Associations between childcare burden during the COVID-19 pandemic with the decline in desired number of children among mothers (*n* = 622).

	Model 1		Model 2	
	Crude	Adjusted	Crude	Adjusted
*Taking care of children during the lockdown*				
Only/mainly me	1.00 (reference)	1.00 (reference)	1.00 (reference)	1.00 (reference)
Half-time me and my partner	0.91 (0.61, 1.36)	0.85 (0.56, 1.31)	0.91 (0.60, 1.36)	0.84 (0.54, 1.31)
Only/mostly my partner or someone else	0.75 (0.45, 1.26)	0.71 (0.41, 1.23)	0.85 (0.50, 1.44)	0.79 (0.45, 1.39)
*COVID-related stress*			**1.80 (1.49, 2.18)**	**1.80 (1.47, 2.21)**
*Taking care of children during the quarantine*				
Not in quarantine with children	1.00 (reference)	1.00 (reference)	1.00 (reference)	1.00 (reference)
Only/mainly me	**1.71 (1.07, 2.72)**	**1.91 (1.16, 3.15)**	1.57 (0.96, 2.54)	**1.78 (1.06, 2.99)**
Half-time me and my partner	1.21 (0.74, 1.97)	1.11 (0.67, 1.86)	1.10 (0.67, 1.83)	1.01 (0.60, 1.73)
Only/mostly my partner or someone else	2.79 (0.56, 14.08)	2.04 (0.36, 11.56)	3.04 (0.55, 16.74)	2.35 (0.37, 14.92)
*COVID-related stress*			**1.79 (1.48, 2.17)**	**1.80 (1.46, 2.21)**

Odds ratios (95% confidence intervals) are reported. Estimates in bold are significant at *p* < 0.05.

Model 1 included the predictor of interest. Taking care of children during the lockdown and during the quarantine were examined in separate models. Model 2 additionally included COVID-related stress. Models with adjusted odds ratios were controlled for age, partnership status, the number of children, education, employment situation, a judgment of own financial situation, change in income during the pandemic, and place of residence.

### Associations between COVID exposure index, COVID-related stress and desired number of children

Table 4 shows the associations between COVID exposure index and COVID-related stress with the decline in the desired number of children among mothers. A dose–response association was observed between the COVID exposure index and a decline in fertility desires: those mothers who had more contacts with SARS-CoV-2 infection were more likely to decrease their desired number of children (adjusted OR = 1.39, 95% CI 1.12, 1.72 for a linear trend; Table 4). Likewise, a dose–response association was found between COVID-related stress and decreased fertility desires: mothers who reported being more stressed due to the pandemic were especially likely to decrease their desired number of children (Table 4).

**Table 4:** Associations between COVID exposure index and COVID-related stress with the decline in the desired number of children among mothers (*n* = 622).

Predictors	Crude	Adjusted
*COVID exposure index*		
0 (no exposure)	1.00 (reference)	1.00 (reference)
1	1.81 (0.64, 5.10)	2.25 (0.75, 6.75)
2	2.29 (0.85, 6.18)	**2.24 (1.03, 8.39)**
3+ (exposure to 3 or 4 items)	**3.03 (1.13, 8.12)**	**3.84 (1.35, 10.94)**
Linear trend for COVID-19 exposure index*	**1.35 (1.10, 1.65)**	**1.39 (1.12, 1.72)**
*COVID-related stress*		
0 (lowest stress quartile)	1.00 (reference)	1.00 (reference)
1	1.43 (0.79, 2.60)	1.48 (0.90, 2.74)
2	**2.00 (1.17, 3.43)**	**1.94 (1.12, 3.39)**
3 (highest stress quartile)	**3.72 (2.19, 6.30)**	**3.78 (2.17, 6.60)**
Linear trend for COVID-related stress*	**1.81 (1.49, 2.18)**	**1.81 (1.48, 2.22)**

Odds ratios (95% confidence intervals) are reported. Estimates in bold are significant at *p* < 0.05. All predictors were examined in separate models. Models were adjusted for age, partnership status, the number of children, education, employment situation, own financial situation, change in income during the pandemic, and place of residence. *Linear trends for the COVID exposure index and for the COVID-related stress variable were examined in separate models.

### Moderating role of COVID-related stress in the association between childcare burden and fertility desires

We further examined whether the associations between childcare burden and the decline in fertility desires were modified by COVID-related stress. The likelihood ratio test suggests that there is some evidence of an interaction between taking care of children during quarantine and COVID-related stress on fertility desires (*p* = 0.007). In particular, mothers with high levels of COVID-related stress were more likely to decrease their fertility desires regardless of who was taking care of children during the quarantine: OR = 1.52, 95% CI 1.19, 1.95 for mothers not in quarantine, OR = 1.88, 95% CI 1.17, 3.04 for mothers who solely/mainly took care of their children, and OR = 3.42, 95% CI 1.88, 6.23 for mothers who equally shared responsibilities of taking care of children with their partners (no associations was found if a partner or someone else took care of children during the quarantine).

## Discussion

Drawing on survey-based data from Poland collected after the official lockdown response to the COVID-19 pandemic, we observed that almost one-third of mothers decreased their fertility desires. Increased childcare burden was related to a decline in the desired number of children, but only when experienced during quarantines, and not the lockdown. We also found that higher COVID-related stress and increased COVID exposure were related to a decline in fertility desires, regardless of childcare responsibilities during the pandemic. Finally, women who had one child (in comparison to two or more children), whose income decreased during the pandemic and who judged their financial situation as bad, as well as those who studied or combined work with studies, were more likely to decrease their fertility desires during the pandemic.

Partially in line with our hypothesis that a greater childcare burden on mothers during the pandemic would be related to a decline in fertility desires, we found that those mothers who solely or mainly took care of their children during quarantines, but not during the lockdown, were more likely to decrease their desired number of children. This finding aligns with the gender equity framework (Goldscheider et al., 2015; Aassve et al., 2020) suggestion about the increased childcare burden on parents during the COVID-19 pandemic. However, to the best of our knowledge, no previous studies examined the role of childcare burden during the COVID-19 pandemic in the changes in fertility desires, although previous studies showed that parents were more likely to decrease their fertility desires and intentions than childless people (Malicka et al., 2021; Lazzari et al., 2023). Interestingly, we observed different associations between childcare burden and fertility desires depending on whether mothers reported taking care of children during quarantines or lockdowns. This discrepancy may stem from the fact that these two groups of mothers differ. On the one hand, all mothers in Poland were affected by the lockdown restrictions and a lack of support during that period in a similar way. On the other hand, additional quarantine restrictions were imposed on mothers who either had positive COVID-19 test results themselves or whose close family members tested positive for COVID-19 (in other words, whose COVID exposure index was greater). These additional restrictions may have led to the increased COVID-related stress among mothers. Given that stress is related to a decrease in fertility desires (Kahn et al., 2021), it is plausible that additional stress due to quarantine on top of the lockdown may have a greater effect on fertility desires.

We found that higher COVID-related stress *per se* was associated with a decline in the desired number of children among mothers, regardless of childcare responsibilities during the quarantine. Especially among women, increased stress and worries due to the pandemic were related to more negative emotional experiences, including higher psychological distress and fear (Alsharawy et al., 2021). Higher stress levels and worries can also trigger anxiety or depression and, in turn, cause a more negative outlook for the future, leading to decreased fertility desires. Furthermore, we also found that greater COVID exposure was associated with a decline in the desired number of children. In line with this, a study from Iran found that those women who were hospitalized during the pandemic had lower odds of having positive attitudes toward fertility (Afshari et al., 2022). One possible explanation for these findings is that women who were exposed to the SARS-CoV-2 infection might be more fearful about the negative consequences of the virus on their health and fetal health compared to those women who were not exposed to the SARS-CoV-2 infection. In fact, several studies showed that higher concerns about the impact of SARS-CoV-2 infection on health were related to the postponement of childbearing (Micelli et al., 2020; Zhu et al., 2020; Flynn et al., 2021).

It is not surprising that mothers whose financial situation worsened reported lower fertility desires compared to mothers who were in a better financial situation or whose income remained the same or increased during the pandemic. This finding corresponds with previous studies showing that less financially secure women decrease their fertility preferences (Lindberg et al., 2020; Luppi et al., 2020; Kahn et al., 2021; Lin et al., 2021; Sienicka et al., 2021; Tan et al., 2021). Lack of financial resources leads to a lack of stability in life, which was reported as one of the major reasons to postpone having children among people of childbearing age (Savelieva et al., 2022). Moreover, we also found that unfinished studies or combining work with studies were related to a decrease in fertility desires among mothers. Previous studies showed that employment insecurity delays family formation (Schmitt, 2021). Therefore, our results are in line with the notion that an uncertain life situation is associated with the postponement of childbearing.

## Limitations

This study has some limitations that should be considered when interpreting the results. First and foremost, the study sample is not representative of the general population since most of the mothers were highly educated and came from larger towns and cities; therefore, our findings may not be generalizable to the general population. Nevertheless, our results are in line with previous studies covering more diverse populations (Lindberg et al., 2020; Luppi et al., 2020; Zhu et al., 2020; Kahn et al., 2021; Afshari et al., 2022).

Second, this is a cross-sectional study that examined changes in fertility desires due to the COVID-19 pandemic but did not test the actual fertility behavior. Follow-up studies are needed to further examine whether changes in fertility preferences will result in changes in fertility behavior.

Finally, the question about the number of children before the pandemic has a retrospective character, hence, the responses could be influenced by the current state of participants.

## Conclusion

Our study adds to the previous literature on the effects of the COVID-19 pandemic on fertility preferences by showing that a higher childcare burden during quarantines is related to a lower desired number of children among mothers. Also, higher COVID-related stress and COVID exposure were associated with a decreased wish to have children, regardless of childcare responsibilities during the pandemic. Regarding the socio-demographic characteristics of mothers, less financially secure mothers were more likely to decrease their fertility desires. In terms of practical implications, our findings point to several recommendations for policymakers to help mitigate the effects of crises on people’s fertility plans: (1) it would be beneficial to support women who solely or mainly took care of their children to ease their childcare burden; (2) to provide psychological support for parents to lower pandemic-related stress; (3) to financially support the least secure groups of parents.

## Data availability statement

The raw data supporting the conclusions of this article will be made available by the authors, without undue reservation.

## Ethics statement

The studies involving humans were approved by Ethics approval of the study protocol was granted by the Bioethics Committee at Jagiellonian University (opinion number 1072.6120325.2020, obtained on 25.11.2020). The studies were conducted in accordance with the local legislation and institutional requirements. The participants provided their written informed consent to participate in this study.

## Author contributions

UM and IN designed the study. KG performed the statistical analyses and drafted the manuscript. All authors commented on and accepted the final version of the manuscript.

## References

[ref1] Aassve A. Cavalli N. Mencarini L. Plach S. Livi Bacci M. (2020). The COVID-19 pandemic and human fertility. Science 369, 370–371. 32703862 10.1126/science.abc9520

[ref2] Aassve A. Cavalli N. Mencarini L. Plach S. Sanders S. (2021). Early assessment of the relationship between the COVID-19 pandemic and births in high-income countries. Proc. Natl. Acad. Sci. 118:e2105709118. doi: 10.1073/pnas.2105709118, PMID: 34462356 PMC8433569

[ref3] Afshari P. Abedi P. Beheshtinasab M. (2022). Fertility decision of Iranian women during the COVID-19 pandemic and home quarantine: a cross-sectional study in Iran. Front. Psychol. 13:993122. doi: 10.3389/fpsyg.2022.993122, PMID: 36457913 PMC9707858

[ref4] Alsharawy A. Spoon R. Smith A. Ball S. (2021). Gender differences in fear and risk perception during the COVID-19 pandemic. Front. Psychol. 12:689467. doi: 10.3389/fpsyg.2021.689467, PMID: 34421741 PMC8375576

[ref5] Arpino B. Luppi F. Rosina A. (2021). Changes in fertility plans during the COVID-19 pandemic in Italy: the role of occupation and income vulnerability. SocArXiv. doi: 10.31235/osf.io/4sjvm

[ref6] Baiocco R. Laghi F. (2013). Sexual orientation and the desires and intentions to become parents. J. Fam. Stud. 19, 90–98. doi: 10.5172/jfs.2013.19.1.90

[ref7] Buber-Ennser I. Setz I. Riederer B. (2023). Not even a pandemic makes them change their family plans: the impact of COVID-19 on fertility intentions in Austria. Popul. Dev. Rev. doi: 10.1111/padr.12555

[ref8] Cohen P. N. (2021). Baby bust: falling fertility in US counties is associated with COVID-19 prevalence and mobility reductions. SocArXiv. doi: 10.31235/osf.io/qwxz3

[ref9] Del Boca D. Oggero N. Profeta P. Rossi M. (2020). Women’s and men’s work, housework and childcare, before and during COVID-19. Rev. Econ. Househ. 18, 1001–1017. doi: 10.1007/s11150-020-09502-1, PMID: 32922242 PMC7474798

[ref10] Dunatchik A. Gerson K. Glass J. Jacobs J. A. Stritzel H. (2021). Gender, parenting, and the rise of remote work during the pandemic: implications for domestic inequality in the United States. Gend. Soc. 35, 194–205. doi: 10.1177/08912432211001301, PMID: 35599685 PMC9122150

[ref11] Emery T. Koops J. C. (2022). The impact of COVID-19 on fertility behaviour and intentions in a middle income country. PLoS One 17:e0261509. doi: 10.1371/journal.pone.0261509, PMID: 34990459 PMC8735619

[ref12] Flynn A. C. Kavanagh K. Smith A. D. Poston L. White S. L. (2021). The impact of the COVID-19 pandemic on pregnancy planning behaviors. Women’s Heal. Reports 2, 71–77. doi: 10.1089/whr.2021.0005 PMC8006747 33786533

[ref13] Goldscheider F. Bernhardt E. Lappegård T. (2015). The gender revolution: a framework for understanding changing family and demographic behavior. Popul. Dev. Rev. 41, 207–239. doi: 10.1111/j.1728-4457.2015.00045.x

[ref9001] Irani M. Bashtian M. Soltani N. Khabiri F. (2022). Impact of COVID-19 on mental health of infertile couple: A rapid systematic review. J. Educ. Health Promot. 11:404. doi: 10.4103/jehp.jehp_1655_21, PMID: 36824404 PMC9942163

[ref14] Kahn L. G. Trasande L. Liu M. Mehta-Lee S. S. Brubaker S. G. Jacobson M. H. (2021). Factors associated with changes in pregnancy intention among women who were mothers of young children in New York City following the COVID-19 outbreak. JAMA Netw. Open 4:e2124273. doi: 10.1001/jamanetworkopen.2021.24273, PMID: 34524437 PMC8444024

[ref15] Lazzari E. Reimondos A. Gray E. (2023). Did the COVID-19 pandemic affect fertility desires in Australia? Understanding why people changed their attitudes towards having a first or additional child. Popul. Dev. Rev. doi: 10.1111/padr.12549

[ref16] Li R. Yin T. Fang F. Li Q. Chen J. Wang Y.. (2020). Potential risks of SARS-CoV-2 infection on reproductive health. Reprod. Biomed. Online 41, 89–95. doi: 10.1016/j.rbmo.2020.04.018, PMID: 32466994 PMC7192111

[ref17] Lin T. K. Law R. Beaman J. Foster D. G. (2021). The impact of the COVID-19 pandemic on economic security and pregnancy intentions among people at risk of pregnancy. Contraception 103, 380–385. doi: 10.1016/j.contraception.2021.02.001, PMID: 33587906 PMC9748659

[ref18] Lindberg L. D. Mueller J. Kirstein M. VandeVusse A. The continuing impacts of the COVID-19 pandemic in the United States: Findings from the 2021 Guttmacher survey of reproductive health experiences. New York Guttmacher Institute (2021).

[ref19] Lindberg L. D. VandeVusse A. Mueller J. Kirstein M. Early impacts of the COVID-19 pandemic: Findings from the 2020 Guttmacher survey of reproductive health experiences. New York: Guttmacher Institute (2020).

[ref20] Luppi F. Arpino B. Rosina A. (2020). The impact of COVID-19 on fertility plans in Italy, Germany, France, Spain, and the United Kingdom. Demogr. Res. 43, 1399–1412. doi: 10.4054/DemRes.2020.43.47

[ref21] Malicka I. Mynarska M. Świderska J. (2021). Perceived consequences of the COVID-19 pandemic and childbearing intentions in Poland. J. Fam. Res. 33, 674–702. doi: 10.20377/jfr-666

[ref22] Marteleto L. J. Dondero M. (2021). Navigating women’s reproductive health and childbearing during public health crises: Covid-19 and Zika in Brazil. World Dev. 139:105305. doi: 10.1016/j.worlddev.2020.105305, PMID: 35495087 PMC9053522

[ref23] Mas A. Pallais A. (2020). Alternative work arrangements. Annu. Rev Econom. 12, 631–658. doi: 10.1146/annurev-economics-022020-032512

[ref24] Micelli E. Cito G. Cocci A. Polloni G. Russo G. I. Minervini A.. (2020). Desire for parenthood at the time of COVID-19 pandemic: an insight into the Italian situation. J. Psychosom. Obstet. Gynecol. 41, 183–190. doi: 10.1080/0167482X.2020.1759545, PMID: 32379999

[ref25] Miller W. B. (2011). Differences between fertility desires and intentions: implications for theory, research and policy. Vienna Yearb. Popul. Res. 9, 75–98. doi: 10.1553/populationyearbook2011s75

[ref26] Naya C. H. Saxbe D. E. Dunton G. F. (2021). Early effects of the COVID-19 pandemic on fertility preferences in the United States: an exploratory study. Fertil. Steril. 116, 1128–1138. doi: 10.1016/j.fertnstert.2021.05.092, PMID: 34325920 PMC8421245

[ref27] Nitsche N. Jasilioniene A. Nisén J. Li P. Kniffka M. S. Schöley J.. (2022). Pandemic babies? Fertility in the aftermath of the first COVID-19 wave across European regions. MPIDR Working Paper WP 2022–0271. doi: 10.4054/MPIDR-WP-2022-027

[ref28] Pashazadeh Kan F. Raoofi S. Rafiei S. Khani S. Hosseinifard H. Tajik F.. (2021). A systematic review of the prevalence of anxiety among the general population during the COVID-19 pandemic. J. Affect. Disord. 293, 391–398. doi: 10.1016/j.jad.2021.06.073, PMID: 34246947 PMC8712560

[ref29] Philipov D. Bernardi L. (2012). Concepts and operationalisation of reproductive decisions implementation in Austria, Germany and Switzerland. Comp. Popul. Stud. 36, 495–530. doi: 10.12765/CPoS-2011-14

[ref30] Pomar L. Favre G. de Labrusse C. Contier A. Boulvain M. Baud D. (2022). Impact of the first wave of the COVID-19 pandemic on birth rates in Europe: a time series analysis in 24 countries. Hum. Reprod. 37, 2921–2931. doi: 10.1093/humrep/deac215, PMID: 36228661 PMC9619770

[ref31] Safdari Dehcheshmeh F. Noroozi M. Memar S. Taleghani F. (2023). Childbearing decisions and related factors in the COVID-19 pandemic: a narrative review study. J. Educ. Health Promot. 12, 1–10. doi: 10.4103/jehp.jehp_594_22 37113433 PMC10127467

[ref32] Savelieva K. Jokela M. Rotkirch A. (2022). Reasons to postpone childbearing during fertility decline in Finland. Marriage Fam. Rev. 59, 253–276. doi: 10.1080/01494929.2022.2083283

[ref33] Schmitt C. (2021). The impact of economic uncertainty, precarious employment, and risk attitudes on the transition to parenthood. Adv. Life Course Res. 47:100402. doi: 10.1016/j.alcr.2021.100402, PMID: 36695145

[ref34] Shevlin M. Hyland P. Ben-Ezra M. Karatzias T. Cloitre M. Vallières F.. (2020). Measuring ICD-11 adjustment disorder: the development and initial validation of the international adjustment disorder questionnaire. Acta Psychiatr. Scand. 141, 265–274. doi: 10.1111/acps.13126, PMID: 31721147

[ref35] Sienicka A. Pisula A. Pawlik K. K. Kacperczyk-Bartnik J. Bartnik P. Dobrowolska-Redo A.. (2021). The impact of COVID-19 pandemic on reproductive intentions among the polish population. Ginekol. Pol. 93, 345–350. doi: 10.5603/GP.a2021.0135, PMID: 34263917

[ref36] StataCorp LLC Stata statistical software: release 17. StataCorp LLC, College Station, TX (2021).

[ref37] Tan P. L. Ryan J. Lim-Soh J. W. (2021). Epidemics and fertility change: responses to Zika and COVID-19 in Singapore. SSRN Electron. J. doi: 10.2139/ssrn.3919334

[ref38] Vseviov H. Puur A. Gortfelder M. (2023). Fertility intentions and sexual orientation: evidence from the 2020 youth survey in Estonia. Popul. Res. Policy Rev. 42:15. doi: 10.1007/s11113-023-09773-3

[ref39] Wang Y. Gozgor G. Lau C. K. M. (2022). Effects of pandemics uncertainty on fertility. Front. Public Heal. 10:854771. doi: 10.3389/fpubh.2022.854771 PMC9468419 36111195

[ref40] Zhu C. Wu J. Liang Y. Yan L. He C. Chen L.. (2020). Fertility intentions among couples in Shanghai under COVID-19: a cross-sectional study. Int. J. Gynecol. Obstet. 151, 399–406. doi: 10.1002/ijgo.13366 PMC9087787 32880942

