# Supplementary material for: Childcare burden and changes in fertility desires of mothers during the COVID-19 pandemic
Source: Front Psychol. 2023 Nov 30;14:1243907. doi: 10.3389/fpsyg.2023.1243907 (PMC10723901; doi:10.3389/fpsyg.2023.1243907)
Supplement: Supplementary file 1 [file Table_1.DOCX]

**Supplementary materials**

**Childcare burden and changes in fertility desires of mothers during the COVID-19 pandemic**

**Appendix Table 1.** The measure of COVID-related stress.

“The following statements reflect the problems that people sometimes experience in connection with stressful life events, such as the current epidemiological situation. When reflecting on your COVID-19 experience, please indicate how much you agree with each of the following statements” (response scale: 1= not at all, 2 = a little, 3 = moderately, 4 = a lot, 5 = almost all the time):

1. I worry a lot more since the COVID-19 epidemic started.
2. I can't stop thinking about COVID-19.
3. Since the COVID-19 epidemic began, I am often concerned about what might happen in the future.
4. Since the COVID-19 epidemic began, I have had a hard time adjusting to life.
5. Since the beginning of the COVID-19 epidemic, I have had great difficulty calming down and relaxing.
6. Since the COVID-19 epidemic began, it has been hard for me to achieve a state of inner peace.

**Appendix Table 2**. Results from the latent confirmatory factor analysis for the scale of COVID-related stress.

| **Translation of the item into English** | **β** | ***p-value*** |
| --- | --- | --- |
| I worry a lot more since the COVID-19 epidemic started | .66 | <0.001 |
| I can't stop thinking about COVID-19 | .61 | <0.001 |
| Since the COVID-19 epidemic began, I am often concerned about what might happen in the future | .66 | <0.001 |
| Since the COVID-19 epidemic began, I have had a hard time adjusting to life | .81 | <0.001 |
| Since the beginning of the COVID-19 epidemic, I have had great difficulty calming down and relaxing | .79 | <0.001 |
| Since the COVID-19 epidemic began, it has been hard for me to achieve a state of inner peace | .84 | <0.001 |

*Note*. The response scale ranges from 1 (*not at all*) to 5 (*almost all the time*).

Model fit: χ² (4) = 12.72, p = 0.013, CFI = 0.996, TLI = 0.986, and RMSEA = 0.060 (0.025, 0.098). Model fit was assessed using the Comparative Fit Index (CFI), Tucker-Lewis Index

(TLI), and the Root Mean Square Error of Approximation (RMSEA) from the confirmatory factor analysis using structural equation modelling in Stata 17.0. CFI and TLI values closer to .95 and RMSEA equal to or under .06 provide reliable evidence of acceptable model fit^1^.

**Reference:**

1. Hu, L. & Bentler, P. M. Cutoff criteria for fit indexes in covariance structure analysis: Conventional criteria versus new alternatives. *Struct. Equ. Model. A Multidiscip. J.* **6**, 1–55 (1999).

**Appendix Table 3.** Associations between socio-demographic characteristics with a decline in the desired number of children from a multivariable model (n = 622).

|  | OR | 95% CI |
| --- | --- | --- |
| *Age* | 1.02 | 0.98, 1.08 |
| *Partnership status* |  |  |
| Single | 1.00 (reference) | |
| In a stable relationship | 1.78 | 0.45, 7.11 |
| Married | 2.14 | 0.58, 7.82 |
| *Number of children* |  |  |
| One | 1.00 (reference) | |
| Two | **0.61** | **0.40, 0.93** |
| Three or more | **0.44** | **0.23, 0.84** |
| *Education* |  |  |
| Secondary or lower | 1.00 (reference) | |
| Lower tertiary | 0.52 | 0.24, 1.11 |
| Upper tertiary | 0.68 | 0.36, 1.28 |
| *Employment situation* |  |  |
| Not studying or working | 1.00 (reference) | |
| Working and/or studying | **2.17** | **1.05, 4.50** |
| Working | 1.12 | 0.66, 1.88 |
| *Own financial situation* |  |  |
| Ver bad/bad/average | 1.00 (reference) | |
| Good | 0.78 | 0.51, 1.19 |
| Very good | 0.61 | 0.34, 1.11 |
| *Change in income during the pandemic* |  |  |
| No | 1.00 (reference) | |
| Yes, decreased | **2.20** | **1.48, 3.27** |
| Yes, increased | 1.38 | 0.63, 3.05 |
| *Place of residence* |  |  |
| Village | 1.00 (reference) | |
| <20,000-100,000 residents | 1.23 | 0.66, 2.27 |
| 100,000-500,000 residents | 0.82 | 0.42, 1.59 |
| >500,000 residents | 0.96 | 0.56, 1.65 |

*Note*. OR = odds ratio, CI = confidence intervals. Estimates in bold are significant at *p* < 0.05.
